# Limited access to improved drinking water, unimproved drinking water, and toilet facilities among households in Ethiopia: *Spatial and mixed effect analysis*

**DOI:** 10.1371/journal.pone.0266555

**Published:** 2022-04-01

**Authors:** Daniel Gashaneh Belay, Zewdu Andualem

**Affiliations:** 1 Department of Human Anatomy, School of Medicine, College of Medicine and Health Sciences, University of Gondar, Gondar, Ethiopia; 2 Department of Epidemiology and Biostatistics, Institute of Public Health, College of Medicine and Health Sciences, University of Gondar, Gondar, Ethiopia; 3 Department of Environmental and Occupational Health and Safety, Institute of Public Health, College of Medicine and Health Sciences, University of Gondar, Gondar, Ethiopia; The University of the South Pacific, FIJI

## Abstract

**Background:**

Most people in sub-Saharan countries had limited drinking water services and toilet facilities. The collection of water can affect the health of the whole family, particularly children. Therefore this study aims to investigate determinants of limited access to drinking water services and spatial distributions of limited access to drinking water services, unimproved drinking water sources, and toilet facilities among households in Ethiopia.

**Method:**

Cross-sectional collected secondary data analysis was conducted based on the 2016 Ethiopian Demographic and Health Survey (EDHS). A mixed-effect logistic regression model was used for analysis. The total weighted sample of 10, 183 households was included in the analysis. The study population is comprised of all households who had recorded the time taken to fetch improved drinking water during the survey. The primary outcome of this study is the proportion of households that have limited access to drinking water services. Whereas the secondary outcomes are determinants of limited access to drinking water services and spatial distributions of limited drinking water services, unimproved drinking water sources, and toilet facilities among households in Ethiopia.

**Results:**

In this study, 39.74% [95% CI: 38.79, 40.69] of households in Ethiopia had limited access to drinking water services. This proportion ranges from 2.64% in Addis Ababa to 57.35% in the Somali region. Household head, education, residence, and regions were associated with limited access to drinking water services. The spatial analysis showed that the eastern part of Amhara, Afar and the Somali region predicted the highest limited drinking water services. The unimproved drinking water source was detected in the Central part of Amhara, Somalia regions. Almost all regions except Addis Ababa and Diredewa predicted the highest prevalence of unimproved toilet facilities.

**Conclusion:**

Access to improved drinking water is relatively poor throughout Ethiopia, with some regions experiencing more limited access than others. From individual level variables age, educational level, and sex household head, whereas from community level variables residences, and region were factors significantly associated with access to limited drinking water services. The spatial analysis confirmed that there are inequalities in unimproved drinking water sources, and toilet facilities between regions in Ethiopia. The government of Ethiopia should work to increase the accessibility of improved drinking water at the national level and to narrow the gap in its accessibility between urban and rural residences and between regions. Accessibility to drinking water programs should focus on the disadvantaged group such as non-educated, male head households and living rural residences. All stalk holders should work to improve the quality of drinking water and sanitation facilities.

## Background

Diseases related to poor sanitation contribute to 4.0% of all deaths and 5.7% of the global disease burden [1]. Sub-Saharan Africa remains the region with the largest disease burden from inadequate Water Hygiene and Sanitation (WASH) [2]. Of these, 1.9 million deaths and 123 million disability-adjusted life years (DALYs) could have been prevented with adequate WASH [2]. Worldwide, more than 785 million people have no access to basic water services, more than 884 million people have no safe water to drink and more than 25% (2.5 billion) of the world’s population have no access to basic sanitation [3].

The United Nations (UN) has recognized access to safe drinking water as a fundamental human right, and the Sustainable Development Goals (SDGs) state that this access is crucial for preventing disease and improving human wellbeing [4]. Nonetheless, many people do not have enough water to meet their daily needs. When there is not enough water to wash, people can get infections such as childhood diarrhea, helminthiasis, scabies, and trachoma [5, 6], and also cause infections of the bladder and kidneys [7].

The SDG for water and sanitation, Goal 6, calls for universal and equitable access to safe and affordable drinking water by 2030. The first step is providing everyone with a basic service within a 30-minute round trip, and the long-term goal is to ensure everyone has safe water available at home [8]. But, about 44% of the world’s population must leave their homes to fetch the water they need for drinking and other domestic uses [9]. Most of the households using such “non-networked” water supplies are located in sub-Saharan Africa and southern Asia [9, 10]. In Africa, 40 billion working hours a year are spent collecting clean drinking water [11]. On average, women and girls in developing countries walk 6 kilometers per day, which takes more than 15 hours per week [12]. Women and children are principally responsible and main water carriers in low-income countries, often spending more than one hour per water collection trip and making multiple trips per day [13].

The collection of water can affect the health of the whole family, particularly children. When water is not available at home, even if it is collected from a safe source, the fact that it has to be transported and stored increases the risk that it is faecally contaminated by the time it is drunk. This in turn increases the risk of diarrheal disease, which is the fourth leading cause of death among under-five children [8].

Even though water supply and sanitation facilities are showing an increasing pattern in Ethiopia, still it is the lowest water supply (42%) and sanitation coverage (28%) in sub-Saharan countries [14]. Studies showed about 52.1% of the population has been using unimproved sanitation facilities while 36% of them practiced open defecation [15]. The majority of water-related health impact research has focused on water quality, leaving the relationship between time spent water fetching and improved water sources [16, 17].

Therefore, this study aimed to answer the following research questions. What was the magnitude of limited access to improved drinking water among households in Ethiopia in 2016 EDHS? What are the individual and community level factors which contribute to this magnitude?, and what are the spatial distributions of limited access to improved drinking water services, unimproved drinking water sources, and toilet facilities among households in Ethiopia? This may help policymakers and stakeholders to set policies and strategies and to design intervention programs based on the findings reported from the study.

## Methods

### Study design and data source

The study used population-based cross-sectional survey data from EDHS 2016. Ethiopia is an East African country with 1.1 million Sq. km coverage and the second-most populous country in Africa with an estimated population of 114,963,588 in 2021 [18]. Administratively, Ethiopia is federally decentralized into nine regions (Afar, Amhara, Benishangul Gumuz, Gambelia, Harari, Oromia, Somali, Southern Nations Nationalities and People’s Ethiopia (SNNPE) and Tigray) and two Administrative Cities (Addis Ababa and Diredewa), and two Administrative Cities (Addis Ababa and Dire Dawa). Regions are divided into zones, and zones, into administrative units called districts. Each district is further subdivided into the lowest administrative unit, called Kebele. Kebele is also subdivided into census enumeration areas (EAs), which are convenient for the implementation of census. The EDHS used a stratified two-stage cluster sampling technique selected in two stages using the 2007 Population and Housing Census (PHC) as a sampling frame. Stratification was achieved by separating each region into urban and rural areas. In total, 21 sampling strata have been created. In the first stage, 645 EAs (202 in the urban area) were selected with probability selection proportional to the EA size and independent selection in each sampling stratum. In the second stage, on average, 28 households have been systematically selected [19].

### Study population

A total of 16,650 households were studied in EDHS 2016. Since all households have records, for the spatial analysis of unimproved drinking water sources, and toilet facilities, all 16,650 households were taken. Whereas, for mixed effect and spatial analysis of limited access to improved drinking water source study, only households which had improved drinking water source and had recorded for the time taken to fetch improved drinking water during the survey were the study population. Therefore, 6,371 households that had unimproved drinking water sources and 121 households that respond as don’t know for some independent variables were excluded. Finally a total weighted sample of 10, 183 households were included.

### Study variables

The outcome variables of the study were the round trip time to obtain improved drinking water services from the households which were measured in minutes. A household is considered as limited access to improved drinking water service if it obtained drinking water source greater than 30 minutes roundtrip (half of an hour). The independent variables considered for this study were categorized as individual level and community level variables. From individual level variables, socio-demographic factors of the household head such as; age, sex, marital status, education attainment, household family size, person fetching water, source of drinking water, household wealth index, and media exposure status were included. Media exposure status is created from the frequency of watching TV, listening to the radio and reading newspapers or magazines, and if a woman has at least one yes, she has considered having media exposure. The wealth index was already calculated by the “DHS program” using the Gini coefficient and only regrouped the five categories into three: poorest and poorer as poor, middle as middle and richest, and richer as rich [19]. On the other hand, community level variables such as place of residence, and region were considered.

### Operational definitions

#### Limited drinking water services

Drinking water from an improved source for which collection time takes more than 30 minutes for a round trip, including queuing [20].

#### Basic drinking water services

Drinking water from an improved source, provided collection time is less than 30 minutes for a round trip, including queuing [20].

#### Unimproved sources of drinking water

A household is said to have access to an unimproved drinking water source if it has water unprotected dug well, unprotected spring, tanker truck/cart with a small tank, surface water, and others [21].

**Unimproved types of toilet facilities.** A household is said to have access to unimproved toilet facilities if it has flush to somewhere else, flush don’t know where, pit latrine without slab / open pit, no facility/bush/field, bucket toilet, hanging toilet/latrine and other and shared flush/pour flush to piped sewer systems, septic tanks or pit latrines, ventilated improved pit latrines, compositing toilets or pit latrines with slabs [22].

### Data processing and analysis

The data was accessed in Stata format after being registered as an authorized user. STATA version 14 was used for data cleaning and analysis. The “Household Record (HR)” data set was used. The data were weighted using sampling weight before any statistical analysis to restore the representativeness of the survey. Weighted values were used to restore the representativeness of the sample data. Since the overall probability of selection of each household is not a constant, before using the DHS dataset it must be weighted. DHS guideline set four sampling weighting methods and from that, we used household sample weight (hv005) which is the household weight (hv005) multiplied by the inverse of the household response rate for a household in the stratum. Household sample weights are generated by dividing (hv005) by 1,000,000 before use to approximate the number of cases [19].

### Model building

Since the EDHS data has a hierarchical structure where households are nested within a cluster/EAs, which violates the assumption of independence of observations and equal variance across clusters, mixed effect models which include both fixed and random effects were used to assess the clustering effect of limited access to drinking water services.

The fixed effects (a measure of association) were used to estimate the association between the likelihood of limited access to drinking water services and explanatory variables at both households and community levels. In each model of multivariable analysis, the associations between dependent and independent variables were presented using adjusted odds ratios and 95% confidence intervals with a p-value of <0.05. Whereas the random effect (a measure of variation) of the model was checked using cluster (v001) as a random variable to assess the clustering effect and were estimated using the Interclass Correlation Coefficient (ICC), Median Odds Ratio (MOR), and Proportional Change in Variance (PCV). The ICC reveals the variation of limited access to drinking water services between clusters is calculated as; $ICC=\frac{VC}{VC+3.29}*100\%$. The MOR is defined as the median value of the odds ratio between the area at the lowest risk and at the highest risk when randomly picking out two clusters. ${{MOR=e}^{0.95}}^{\sqrt{VC}}$ The PCV shows the variation in limited access to drinking water services among households explained by both household and community level factors. $PCV=\frac{Vnull-VC}{V\;null}*100\%$, Where; Vnull = variance of the initial model, and VC = cluster level variance [23–25].

Generally, in mixed-effect analysis, four models were fitted. The first was the null model containing only the outcome variables which were used to check the variability of limited access to drinking water services in the cluster. The second and the third multilevel models contain household-level variables and community-level variables respectively whereas in the fourth model both household and community level variables simultaneously were fitted with limited access to drinking water services. Model comparison was done using the deviance test and the model with the lowest deviance was selected as the best-fitted model [23–25].

## Spatial analyses

The spatial analysis was conducted for three main environmental health outcome variables; limited drinking water source (round trip collection time ≥ 30 minutes from improved source), unimproved drinking water source, and unimproved toilet facilities.

Spatial autocorrelation (Global Moran’s I) statistic measure was used to assess whether limited drinking water source, unimproved drinking water source, and unimproved toilet facilities among households in Ethiopia were dispersed, clustered, or randomly distributed in Ethiopia using Moran’s I values [26]. The weighted proportion of each of the three variables were exported to Arc GIS 10.7 software and spatial autocorrelation, spatial distribution, and detection of hot spot areas spatial interpolation was done. P-values calculated through a hot spot analysis (Getis-Ord Gi* statistic) of the z-scores allowed the researchers to identify areas with a high proportion of the problems. Spatial scan statistics were employed to determine the geographical locations of statistically significant clusters for those outcomes among households in Ethiopia using Kuldorff’s SaTScan version 9.6 software.

[27] The geostatistical ordinary Kriging spatial interpolation technique is used to predict limited drinking water sources, unimproved drinking water sources, and unimproved toilet facilities among households for unsampled areas based on sampled clusters.

## Result

### Sociodemographic characteristics of the study population

A total weighted 10, 183 households were included in this study, of these nearly three-quarters of the household heads were men 7,395 (72.62%). Most of the study participants 7,328 (71.96%) were living in rural areas and half of the household heads 5,092 (50.00%) had no formal education.

Of the households which have access to improved water sources, two-fifths (39.74%, 95% CI: 38.79, 40.69) had only limited drinking water services, meaning that collection time exceeds 30 minutes for a round trip, including queuing **[Table 1].**

**Table 1:** Socio-demographic characteristics of the study population with limited access to an improved drinking water source in Ethiopia, 2016 EDHS.

Variables	Categories	Limited (%) n = 4,047(39.74%)	Basic (%) n = 6,136 (60.26%)	Total frequency (n = 10, 183)	Percentage (%)
Age of household head (years)	13–30	776(32.72)	1595(67.28)	2,370	23.27
	31–40	1037(40.14)	1547(59.86)	2,584	25.38
	41–56	2,019(47.35)	2,244(52.65)	2,679	26.31
	>57	1173(43.8)	1506(56.20)	2,549	25.04
Sex of household head	Male	2,986 (40.37)	4,409(59.63)	7,395	72.62
	Female	1,061 (38.07)	1,727 (61.93)	2,788	27.38
Educational attainment of household head	No education	2,521 (49.52)	2,570 (50.48)	5,092	50.00
	Primary education	1,205 (39.27)	1,863 (60.73)	3,068	30.14
	Secondary& above	321(15.85)	1,702 (84.15)	2,023	19.86
Marital status of head of household	Married	3172 (42.39)	4310 (57.61)	7,482	73.48
	Not married	875(32.41)	1825 (67.59)	2,701	26.52
House hold family size	1–3	1225 (33.02)	2484 (66.98)	3,709	36.42
	4–6	1881 (41.56)	2646 (58.44)	4,527	44.46
	7 & above	941 (48.34)	1006 (51.66)	1,947	19.12
Media exposure	No	2903 (48.90)	3033 (51.10)	5,936	58.30
	Yes	1144 (26.94)	3103 (73.06)	4,247	41.70
Wealth index	Poor	1539 (56.20)	1200 (43.80)	2,738	26.89
	Middle	952 (49.91)	955 (50.09)	1,906	18.73
	Rich	1556 (28.10)	3981 (71.90)	5,537	54.38
Person who fetch the water	Adult woman	2819 (52.57)	2543 (47.43)	5,361	71.10
	Adult man	384 (52.75)	344 (47.25)	729	9.67
	Female child under 15 years old	555 (57.20)	416 (42.80)	971	12.88
	Male child under 15 years old	192 (56.78)	146 (43.22)	337	4.48
	Other	97(68.45)	45 (31.55)	141	1.88
Residence	Urban	380 (13.32)	2475 (86.68)	2,855	28.04
	Rural	3667 (50.04)	3661 (49.96)	7,328	71.96
Region	Tigray	384 (43.91)	490 (56.09)	874	8.58
	Afar	27 (37.68)	45 (62.32)	71	0.70
	Amhara	1148 (44.05)	1458 (55.95)	2,606	25.59
	Oromia	1250 (35.25)	2296 (64.75)	3,546	34.83
	Somalia	105 (57.36)	78 (42.64)	183	1.80
	B/Gumuz	63 (43.86)	81 (56.14)	144	1.41
	SNNPR	1025 (52.99)	910 (47.01)	1,935	19.00
	Gambelia	9 (24.10)	29 (75.90)	38	0.37
	Harare	5 (15.99)	27 (84.01)	32	0.31
	Addis Ababa	18 (2.64)	669 (97.36)	687	6.75
	Diredewa	12 (18.45)	54 (81.55)	66	0.65

### Mixed effect analysis of factors associated with limited access to drinking water services among households in Ethiopia: Based on 2016 EDHS

In the random effect analysis result of Table 2, the ICC value in the null model showed that about 58% of the variations of limited access to drinking water services among studied households were attributed to the difference at the cluster level, but the other 38% were attributed to individual household factors. The MOR value of 7.56 in the null model revealed the median odds of having limited access to drinking water services between the lowest and the highest clusters were 7.56 times. Furthermore, the PCV valve in the final model (64%) indicates the variation in the limited access drinking water services among study households was explained by both the individual and community level factors simultaneously. Model comparison and fitness were determined using a deviance test. The third model had the lowest deviance (8626) and was thus declared the best fit model **[Table 2].**

**Table 2:** Mixed effect analysis of limited access to drinking water services and its determinants among households in Ethiopia: Based on 2016 EDHS.

Variables	Categories	Null model	Model 1	Model 2	Model 3
			AOR [95% CI]	AOR [95% CI]	AOR [95% CI]
Age of household head (years)	13–30	--------	1.00	--------	1.00
	31–40	-------	1.21[1.027, 1.45]*	------------	1.23[1.04, 1.47]*
	41–56	--------	1.12 [0.94, 1.34]	------------	1.12 [0.94, 1.35]
	>57	--------	0.99 [0.83, 1.19]	------------	1.00 [0.84, 1.21]
Sex of household head	Male	--------	1.00	------------	1.00
	Female	--------	1.36 [1.13, 1.63]	------------	1.34 [1.11, 1.62]*
Educational attainment of household head	No education	-------	1.00	------------	1.00
	Prim. education	------	0.85 [0.74, 0.98]*	------------	0.87 [0.75, 0.99]*
	Secon. & above	------	0.62 [0.49, 0.78]*	------------	0.64 [0.51, 0.80]**
Marital status household head	Married	--------	1.00	------------	1.00
	Not married	--------	0.71[0.58, 0.86]*	------------	0.82 [0.69, 1.02]
Household family size	1–3	-------	1.00	------------	1.00
	4–6	------	0.92 [0.91, 1.22]	------------	1.05 [0.91, 1.21]
	≥7	-------	1.07 [0.88, 1.28]	------------	1.05 [0.87, 1.27]
Media exposure	No	-------	1.00	------------	1.00
	Yes	------	0.97 [0.84, 1.11]	------------	0.98 [0.85, 1.12]
Wealth index	Poor	------	1.00	------------	1.00
	Middle	--------	1.16 [1.04, 1.29]	------------	0.96 [0.82, 1.12]
	Rich	-------	0.94 [0.80, 1.09]	------------	1.05 [0.89, 1.23]
A person who fetch the water	Adult woman	--------	0.62 [0.38, 0.95]	------------	0.62 [0.39, 0.98]
	Adult man	--------	0.71 [0.44, 1.13]	------------	0.71 [0.44, 1.15]
	Female child < 15 years	--------	0.76 [0.47, 1.22]	------------	0.78 [0.48, 1.25]
	Male child < 15 years	--------	0.68 [0.41, 1.14]	------------	0.69 [0.41, 1.15]
	Other	------	1.00	------------	1.00
**Community level variables**					
Residence	Urban	--------	------------	1.00	1.00
	Rural	-------	------------	17.95 [11.1, 29.2]***	1.82 [1.34, 2.23]*
Region	Tigray	--------	------------	1.00	1.00
	Afar	-------	------------	1.57 [0.58, 4.25]	3.65[2.54, 4.76]**
	Amhara	-------	------------	1.08 [0.60, 1.97]	0.88 [0.52, 1.51]
	Oromia	--------	------------	0.54 [0.29, 0.97]**	0.50[0.29, 0.84]**
	Somalia	--------	------------	3.15 [1.49, 6.66]**	2.85 [1.86, 3.78]*
	B/Gumuz	--------	------------	0.83 [0.38, 1.77]	0.63 [0.31, 1.27]
	SNNPR	--------	------------	1.28[0.70, 2.34]	0.96 [0.56, 1.64]
	Gambella	--------	------------	0.40 [0.12, 1.25]	0.23[0.08, 0.67]**
	Harare	--------	------------	0.37 [0.09, 1.51]	0.71 [0.14, 3.50]
	Addis Ababa	--------	------------	0.11 [0.04, 0.30]***	0.27 [0.08, 0.90]*
	Diridawa	--------	------------	0.56 [0.19, 1.72]	0.81 [0.24, 2.80]
**Random effect**					
	Variance	4.55	1.71	2.39	1.62
	ICC	0.58	0.36	0.40	0.33
	MOR	7.56	3.46	4.52	3.34
	PCV	Ref.	0.62	0.47	0.64
**Model comparisons**					
	Deviance	9904	8664	9570	8626
	Mean VIF	------	3.00	1.69	2.50

AOR = adjusted odds ratio; CI = confidence interval

* = P-value < 0.05.

** = P value < 0.01.

*** = P value < 0.001

ICC = Inter cluster correlation coefficient, MOR = Median odds ratio, PCV = proportional change in variance

In the mixed effect multivariable analysis, individual-level variables such as; age, sex, educational attainment, and community level variables such as residence, and region were significantly associated with limited access to improved drinking water service.

Having female household heads have 34% more likely to get limited drinking water sources than households whose heads were male [AOR = 1.34, 95% CI:1.11, 1.62]. Households whose head attained their primary education and secondary & above education have 13% and 36% lower odds of having limited access to an improved drinking water source than households whose head didn’t attain formal education [AOR = 0.87, 95% CI: 0.75, 0.99] and [AOR = 0.64, 95% CI:0.51, 0.80] respectively.

Households in rural areas had 82% greater odds of obtaining water from a limited drinking water source than those in urban areas [AOR = 1.82, 95% CI: 1.34, 2.23]. Households located in the Afar and Somali regions were 3.65 and 2.75 times more likely to have limited drinking water sources [AOR = 3.65, 95% CI:2.54, 4.76] and [AOR = 2.85, 95% CI:1.86, 3.78] respectively. On the other hand, households located in Gambella, Addis Ababa and Oromia were 77%, 73% and 50% times less likely to have limited drinking water sources [AOR = 0.23, 95%CI:0.08, 0.67], [AOR = 0.27, 95%CI: 0.08, 0.90] and [AOR = 0.50, 95% CI: 0.29, 0.84] respectively **[Table 2].**

## Spatial analyses result

### Spatial analysis of limited improved drinking water services among households in Ethiopia, 2016 EDHS

**Fig 1:**
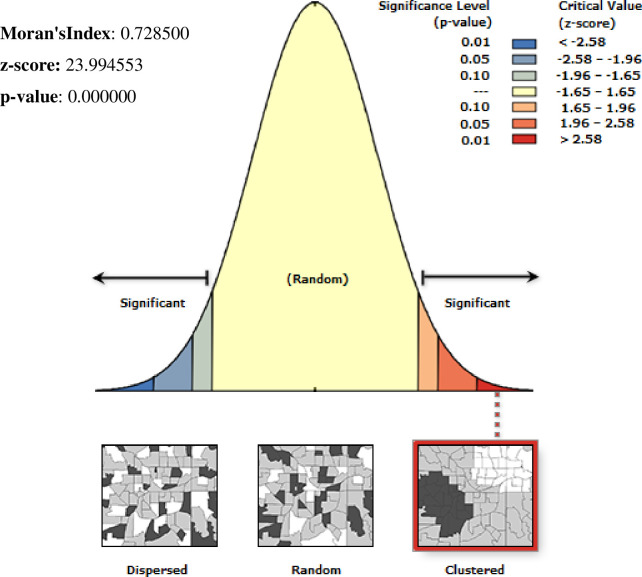
Spatial autocorrelation of limited water service among households in Ethiopia, EDHS 2016, shapefile from 2018 GADM and plotted using ArcMap version 10.7 software.

**Fig 2:**
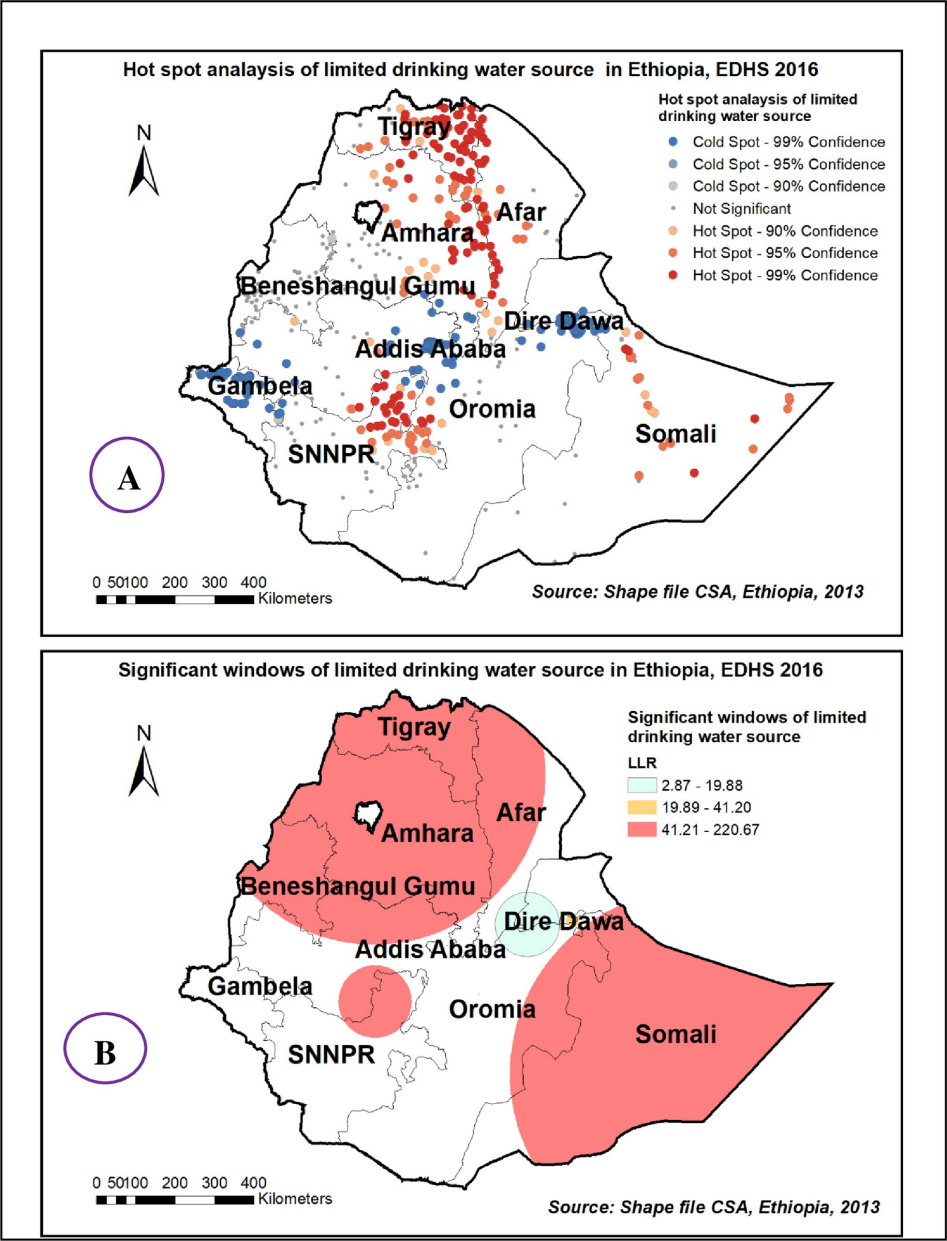
Hot spot areas (A) and significant windows (B) of limited drinking water source in Ethiopia, EDHS 2016, shapefile from 2018 GADM and plotted using ArcMap version 10.7 software.

**Fig 3:**
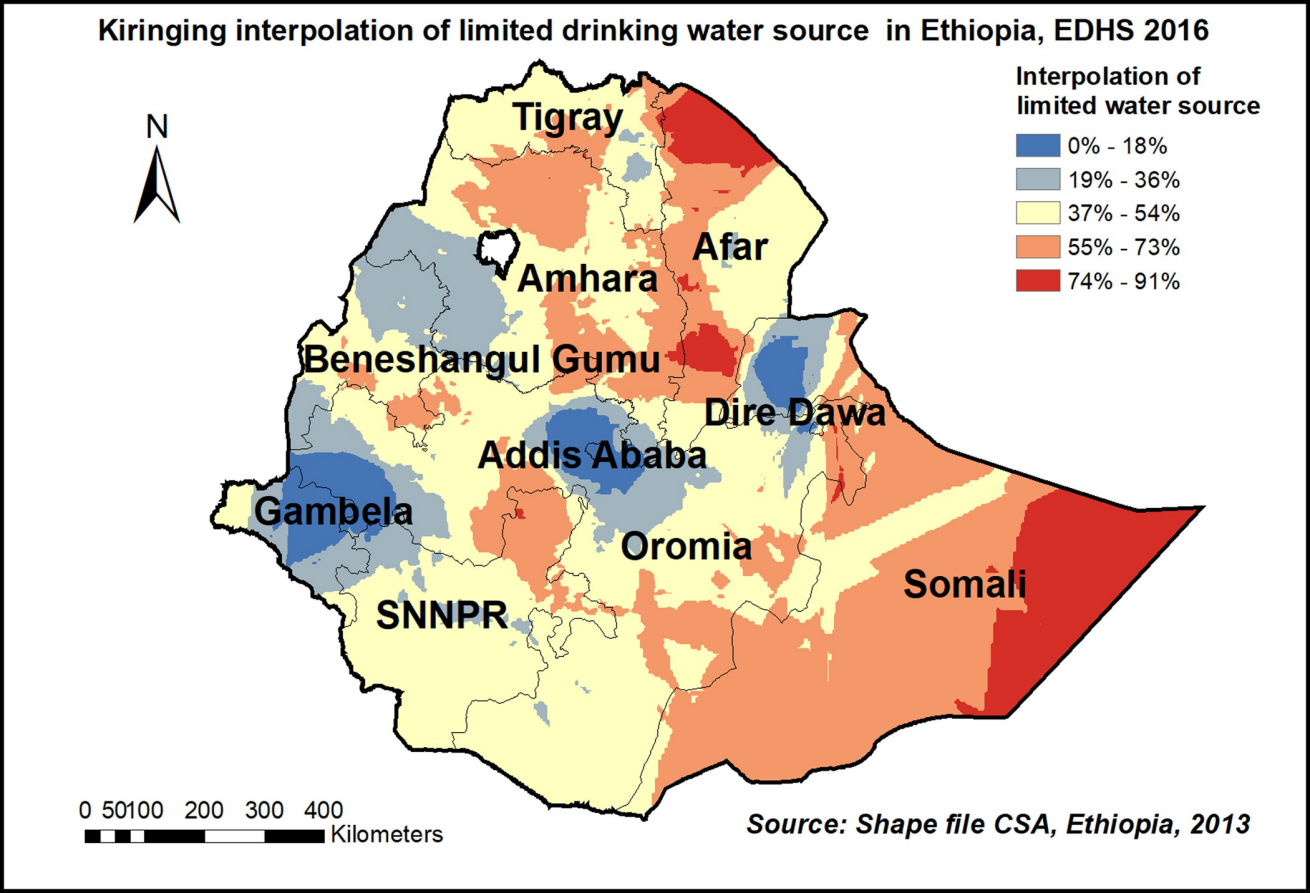
Kiringing interpolation of limited drinking water source in Ethiopia, EDHS 2016, shapefile from 2018 GADM and plotted using ArcMap version 10.7 software.

Spatial distribution of limited drinking water services among households showed significant non-random spatial variation across the country over regions with Global Moran’s I value 0.72(p< 0.0001) **“Fig 1”.** The hot spot areas were detected in western Afar, Eastern Amhara, and Tigray, and the Eastern SNNPE region **“Fig 2A”.** Among 320 most likely clusters having limited water services, 211 of them were primary clusters. These were located in all of Amhara, Tigray, and Benishangul Gumuz, eastern Afar and Somali, as well as on the border between Oromia and SNNPR (centered at 12.910868 N, 37.442592 E, with a 433.35km radius). Households which were found in the SaTScan window were 77% more likely to have limited access to a drinking water source (RR = 1.77, P-value<0.0001) **“Fig 2B & S1 Table**”. The kriging interpolation also revealed that the eastern part of Amhara, Afar, and the Somali region have predicted more limited drinking water sources compared to other regions with the prevalence of high-risk areas ranging from 74% to 91% **“Fig 3”.**

### Spatial analysis unimproved drinking water source among households in Ethiopia, 2016 EDHS

**Fig 4:**
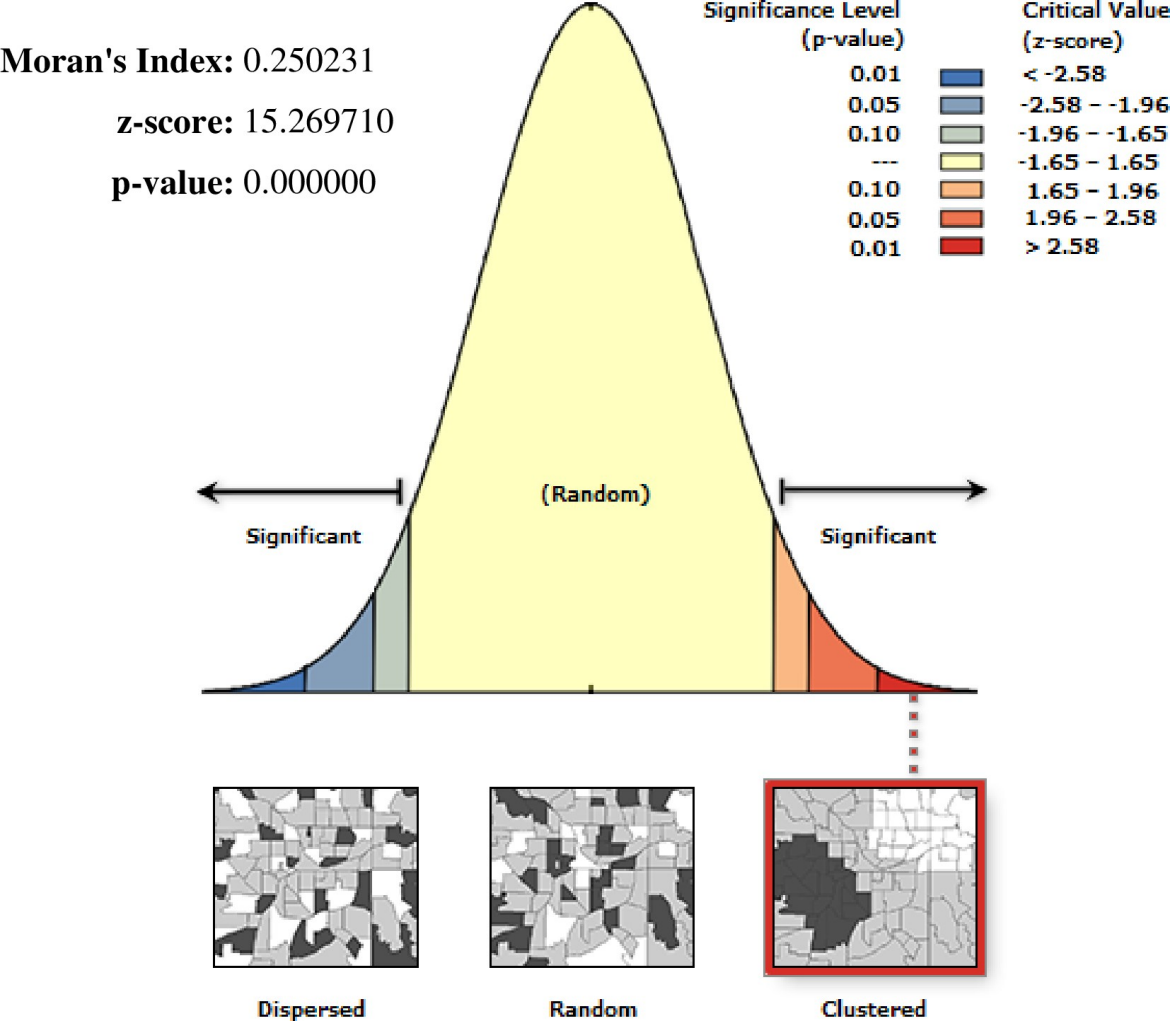
Spatial autocorrelation of unimproved drinking water source among households in Ethiopia, EDHS 2016, shapefile from 2018 GADM and plotted using ArcMap version 10.7 software.

**Fig 5:**
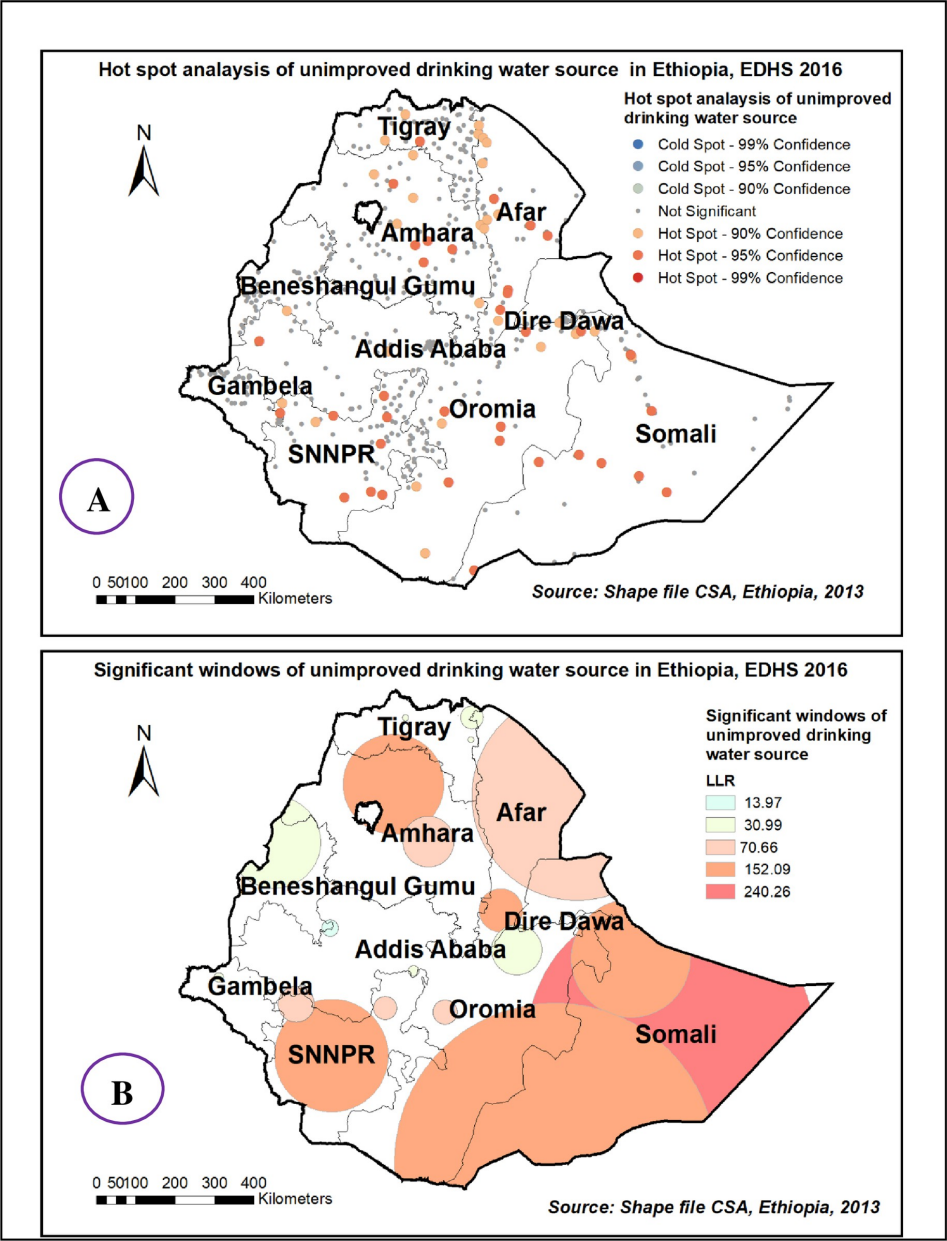
Hot spot areas (A) and significant windows (B) of unimproved drinking water source in Ethiopia, EDHS 2016, shapefile from 2018 GADM and plotted using ArcMap version 10.7 software.

There is a significant non-random spatial variation of unimproved drinking water sources across the country over regions in Ethiopia with Global Moran’s I value 0.25 (p< 0.0001) “**Fig 4**”. High clustering of unimproved drinking water sources was detected in most parts of Amhara, Afar, and Somali region **“Fig 5A”.** Among 186 most significant clusters of having unimproved drinking water sources, 49 of them were primary clusters. These were located in almost entire Somali and Eastern Oromia regions (centered at 6.745502 N, 44.259011 E with a 363.09 km radius). Households which were found in the SaTScan window were 2.03 times more likely to have unimproved a drinking water source (RR = 2.03, P-value <0.0001) “**Fig 5B & S1 Table”**.

**Fig 6:**
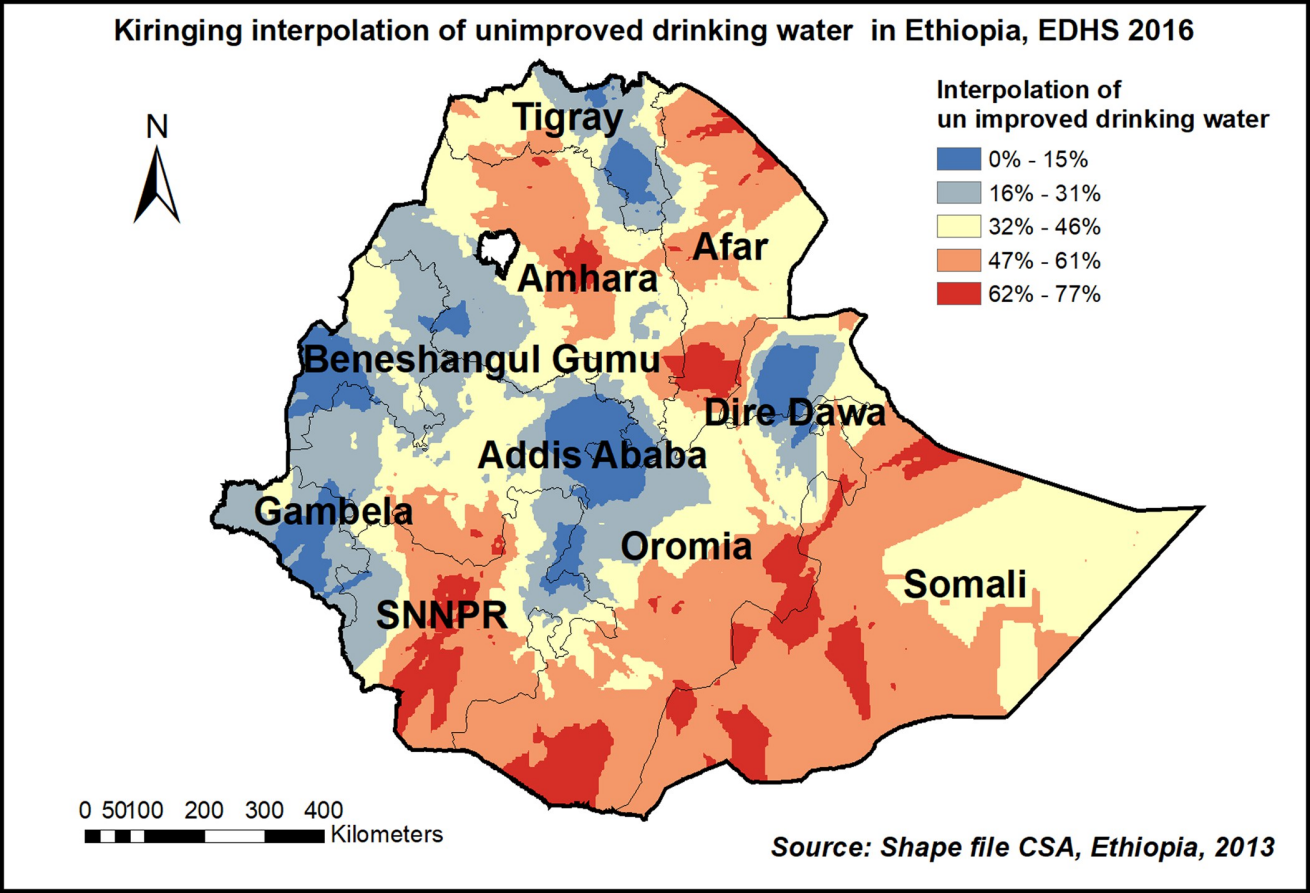
Kiringing interpolation of unimproved drinking water source in Ethiopia, EDHS 2016, shapefile from 2018 GADM and plotted using ArcMap version 10.7 software.

The kriging interpolation also predicts the prevalence of high-risk areas of unimproved drinking water source ranges from 62% to 77% and is detected in the central part of Amhara, Easter Afar, southern SNNPE, Somali and Eastern part of Oromia regions “**Fig 6**”.

### Spatial analysis unimproved toilet facilities among households in Ethiopia, 2016 EDHS

**Fig 7:**
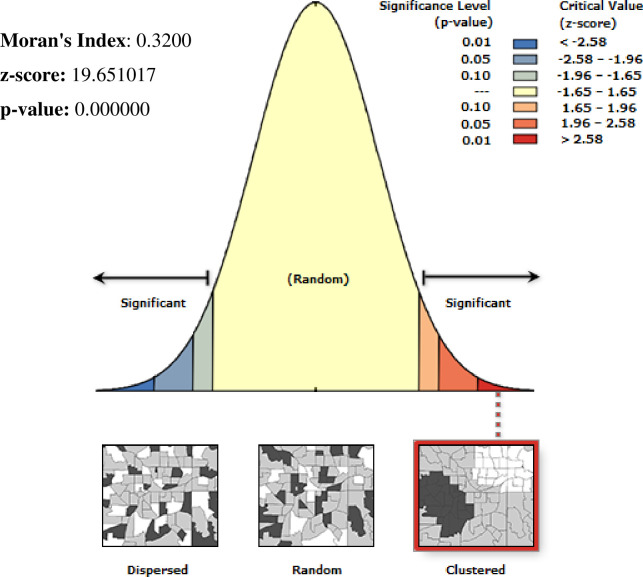
Spatial autocorrelation of unimproved toilet facilities among households in Ethiopia, EDHS 2016, shapefile from 2018 GADM and plotted using ArcMap version 10.7 software.

**Fig 8:**
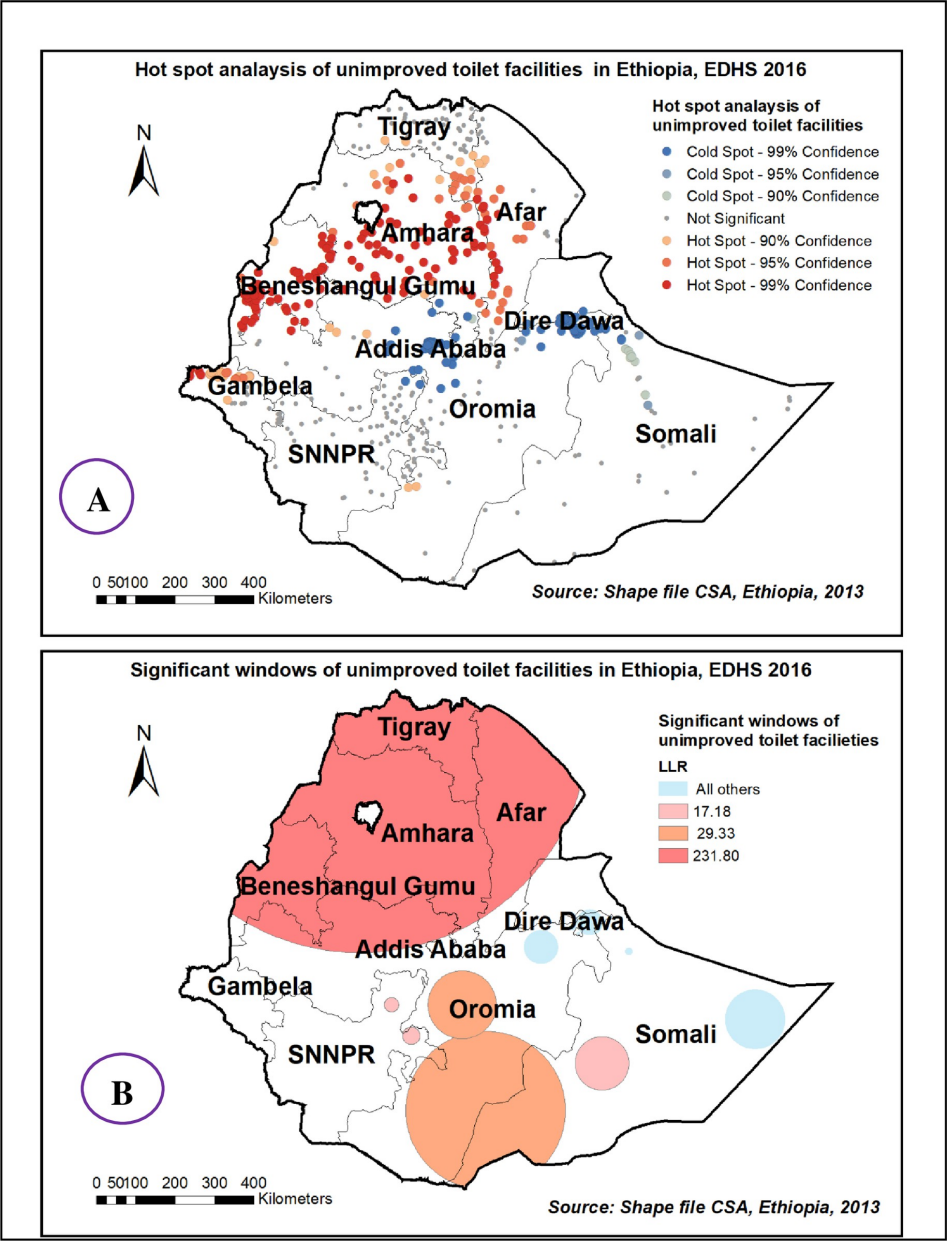
Hot spot areas (A) and significant windows (B) of unimproved toilet facilities in Ethiopia, EDHS 2016, shapefile from 2018 GADM and plotted using ArcMap version 10.7 software.

**Fig 9:**
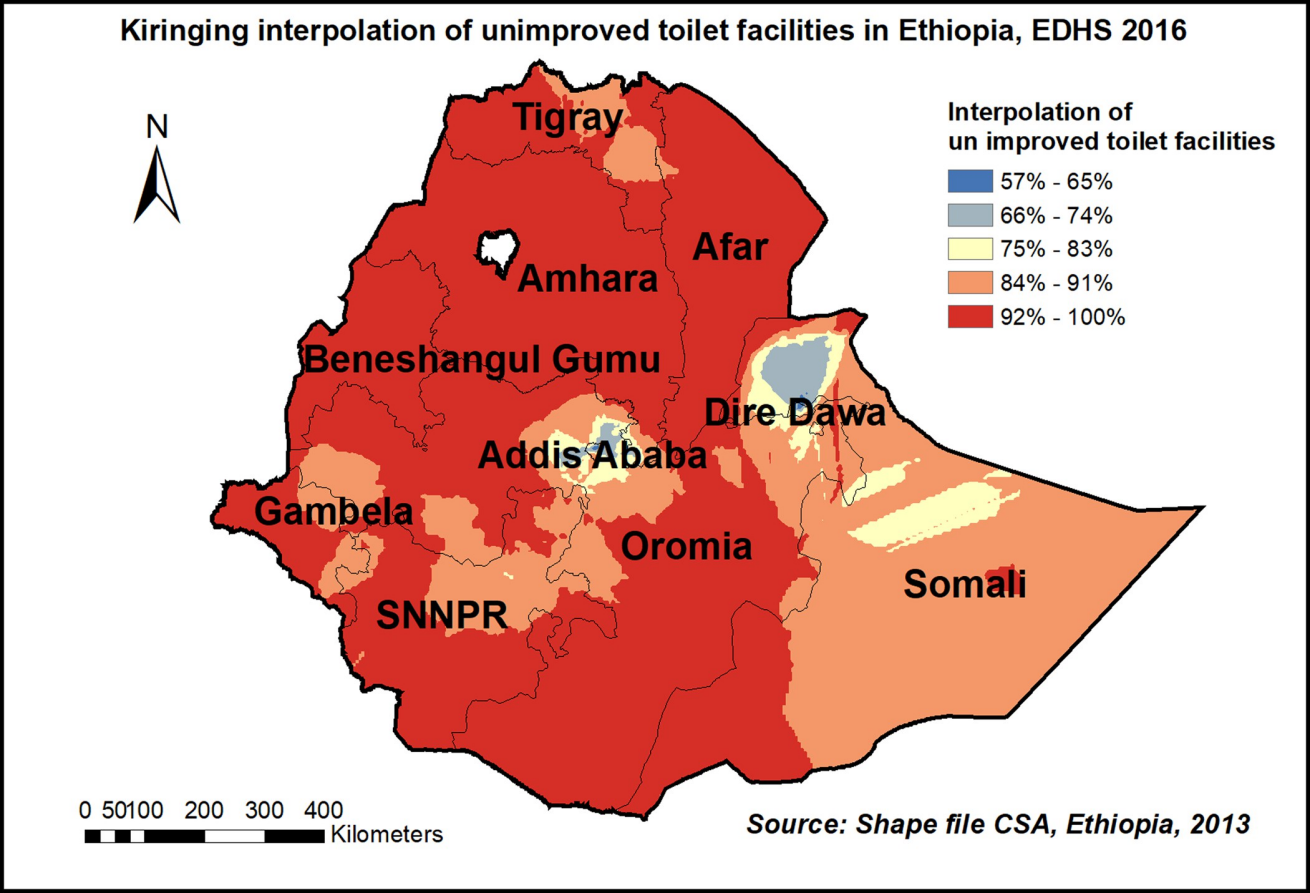
Kriging interpolation of unimproved toilet facilities in Ethiopia, EDHS 2016, shapefile from 2018 GADM and plotted using ArcMap version 10.7 software.

The spatial distribution of unimproved toilet facilities among households showed significant clustered spatial variation across the country with Global Moran’s I value 0.32(p< 0.0001) **“Fig 7”**. Hot spot areas were detected in entire Amhara, Benishangul Gumuz, Afar, and Gambelia region. **“Fig 8A”.** The spatial scan statistics identified 261 significant clusters; among these 238 were primary clusters, which were located in the entire Tigray, Afar, and Amhara, B/Gumuz, and Northern Oromia regions, centered at 14.033877 N, 37.105922 E, with a 578.81 km radius. Households which were found in the SaTScan window were 11% more likely to have unimproved toilet facilities (RR = 1.11, P-value<0.0001) **“Fig 8B & S1 Table”**. The prevalence of high-risk areas of predicted unimproved toilet facilities was extremely high and range from 92% to 100% “**Fig 9**”.

## Discussions

This study found that two-fifths of the households in Ethiopia have access to limited improved drinking water services (39.74%, 95% CI: 38.79, 40.69). Our study finding is higher than the global incidence of access to basic drinking water services (30%) [5]. But, lower than a study conducted in Rwanda [28], Zambia [16], and Kenya [29]. The discrepancy might be due to geographical variation, population growth, and socio-economic status of the countries. This high prevalence of limited drinking water services may contribute to poor attendance at school for children if they are responsible for water collection.

Households whose heads attained primary and secondary & above education have 13% and 36% lower hazard of having limited drinking water services than households whose heads didn’t attain formal education. Our study finding is supported by a previous study [17]. The possible explanation might be those who are educated would have basic knowledge of WASH and they would use different mechanisms to access basic drinking water services. Besides, the educated household head is more likely to be aware of the consequences of poor hygiene and sanitation is more likely to have a reasonable knowledge of how to avert the risks of poor health associated with poor sanitation. Thus, educated households may be more inclined towards making the necessary investments for their households to have better water, hygiene, and sanitation facilities.

In this study, female household heads were 34% more likely to have limited access to drinking water services. This research demonstrates that the sex of the household head plays a role in household access to safe drinking water. The sex of the household head can make a difference in the provision of safe drinking water. In the Ethiopian context, the culture demands that men should be the head of the household except for a few situations where women may take up such responsibility. Household sex plays a role among the determinants of the household choice of a water source. In contrast with our study findings, evidence has shown that male-headed households are less likely to choose an improved source than do female-headed households [30]. On the contrary, in the previous study sex of household heads was not associated with drinking water sources [17].

The odds of obtaining limited water services among households found in rural areas was 82% higher than in urban areas. Our study finding was similar to other previous studies [31, 32]. However, our study finding is opposite to a study finding in Kenya [33]. The scarcity of improved drinking water in rural areas is well documented, as evidenced by the high prevalence of waterborne illnesses such as diarrhea, schistosomiasis, trachoma, and intestinal helminths, which can be directly attributed to contaminated water, poor sanitation, and a lack of hygiene [34]. In addition, the disparity might be due to the government installing houses to house pipe water in urban residences with a small payment in urban settings of Ethiopia.

This study finding revealed that in Ethiopia, there is geographical inequality between regions’ access to basic drinking water services. Regions like Somalia and Afar region had poor access to improved drinking water services, whereas Gambelia, Oromia, and Addis Ababa had better access to improved drinking water services, as compared with the Tigray region. In addition, in the current study the spatial analysis also confirmed that in Addis Ababa, Gambelia, and Benishangul Gumuz region predicted better access to improved drinking water services, in contrast to the eastern part of the Amhara, Afar, and Somali region. Similarly, unimproved drinking water sources predict that the highest unimproved drinking water source was detected in the Central part of Amhara, Easter Afar, SNNPE, Somalia, and the Eastern part of the Oromia regions. On the other side, Addis Ababa, Diredewa, B/Gumuz, Gambelia, and the Tigray region have to predict improved drinking water sources. This might be due to the effectiveness and commitments difference of governmental and non-governmental organizations which work on basic water supplementation.

Regarding unimproved toilet facilities, the current study revealed almost all regions except Addis Ababa and Diredewa predicted the highest prevalence of unimproved toilet facilities. This discrepancy might be that the two cities are the only ones with city administration in Ethiopia and might have modern facilities like pipe water, septic tank, and ventilation which eventually leads to having improved toilet facilities. Generally, understanding the inequality within and between regions, and provide a benchmark for tracking progress, and helping prioritize resource allocation, there is a clear need to develop policy-relevant data on regional inequality and a standardized approach to mapping regional and national coverage in water supply and sanitation [35, 36].

The main strength of this study was the use of the weighted nationally representative data with a large sample which makes it representative at national and regional levels. Therefore, it can be generalized to all households during the study period in Ethiopia. Moreover, the use of a mixed-effect model that took into account the nested nature of the EDHS data and the variability within the community to get a reliable estimate and standard errors. However, it is not free of limitations mainly resulting from the use of secondary data. As some important confounders like the amount of water they get and behavioral factors are missed, the confidence interval for regional categories in mixed-effect analysis becomes wide, due to a large number of categories of regions.

## Conclusions

Access to improved drinking water is relatively poor throughout Ethiopia. From individual level variables age, educational level, and sex of household head, on the other hand from community level variables residences, and region were factors significantly associated with access to limited drinking water services. The spatial analysis confirmed that there is the highest prediction of access to limited drinking water service, unimproved drinking water sources, and toilet facilities within and between regions in Ethiopia. The government of Ethiopia should work to increase the accessibility of improved drinking water at the national level and to narrow the gap in its accessibility between urban and rural residences and between regions. Accessibility to drinking water programs should focus on the disadvantaged group such as non-educated, and male head households and living rural residences. All stalk holders should work to improve the quality of drinking water and sanitation facilities.

## Supporting information

S1 Table Significant spatial primary clusters of limited and unimproved drinking water source and unimproved toilet facilities among households in Ethiopia, EDHS 2016. (PDF)

## Abbreviations

AHR: Adjusted Hazard Ratio
AIC: Akakia Information Criteria
B/Gumuz: Benishangul Gumuz
BIC: Bayesian Information Criteria
CHR: Crude Hazard Ratio
CI: Confidence Interval
CSA: Central Statistical Agency
DALYs: Disability-adjusted life years
DHS: Demographic and Health Survey
EAs: Census Enumeration Areas
EDHS: Ethiopian Demographic Health Survey
GADM: Global Administrative area
SDG: Sustainable Development Goals
SNNPRE: Southern Nations, Nationalities, and Peoples of Ethiopia
WASH: Water Hygiene and Sanitation
