# Correction: Limited access to improved drinking water, unimproved drinking water, and toilet facilities among households in Ethiopia: *Spatial and mixed effect analysis*

**DOI:** 10.1371/journal.pone.0303771

**Published:** 2024-05-09

**Authors:** Daniel Gashaneh Belay, Zewudu Andualem

The second author’s name is spelled incorrectly. The correct name is: Zewudu Andualem. The correct citation is: Belay DG, Andualem Z (2022) Limited access to improved drinking water, unimproved drinking water, and toilet facilities among households in Ethiopia: *Spatial and mixed effect analysis*. PLoS ONE 17(4): e0266555. https://doi.org/10.1371/journal.pone.0266555.
