# Supplementary material for: Limited access to improved drinking water, unimproved drinking water, and toilet facilities among households in Ethiopia: Spatial and mixed effect analysis
Source: PLoS One. 2022 Apr 1;17(4):e0266555. doi: 10.1371/journal.pone.0266555 (PMC8975151; doi:10.1371/journal.pone.0266555)
Supplement: S1 Table — (PDF) [file pone.0266555.s001.pdf]

Supplementary Table 1: Significant spatial primary clusters of limited and unimproved drinking water source and unimproved toilet facilities among households in Ethiopia, EDHS 2016.

	Clusters	Enumeration areas (clusters) detected	Coordinate/radius	Population	Cases	RR	LLR	P-value
Limited drinking water source	1 [211]*	638, 640, 312, 322, 152, 327, 80, 612, 296, 279, 628, 504, 163, 258, 292, 425, 199, 188, 158, 340, 512, 253, 169, 583, 132, 551, 73, 431, 52, 98, 181, 255, 542, 268, 516, 584, 156, 579, 456, 528, 66, 575, 636, 167, 259, 78, 382, 597, 400, 627, 361, 590, 538, 81, 602, 403, 424, 300, 429, 392, 415, 355, 24, 136, 84, 541, 481, 143, 401, 430, 160, 386, 604, 120, 548, 45, 109, 591, 237, 94, 550, 449, 515, 461, 605, 97, 478, 351, 615, 384, 220, 3, 442, 479, 498, 79, 226, 129, 89, 128, 200, 455, 623, 421, 176, 99, 341, 598, 375, 298, 206, 404, 511, 488, 249, 196, 533, 474, 246, 354, 130, 559, 496, 410, 127, 413, 172, 616, 482, 460, 117, 192, 332, 344, 235, 36, 362, 263, 10, 585, 617, 229, 531, 103, 189, 241, 150, 494, 256, 611, 134, 18, 389, 183, 571, 350, 267, 345, 137, 191, 218, 364, 35, 599, 184, 244, 254, 348, 510, 368, 55, 572, 320, 310, 423, 547, 457, 88, 637, 569, 570, 276, 65, 620, 517, 427, 124, 324, 161, 205, 399, 334, 621, 294, 209, 70, 283, 335, 499, 178, 280, 409, 407, 563, 295, 349, 285, 102, 234, 595, 201	12.910868 N, 37.442592 E / 433.35 km	3555	1687	1.77	220.5	<0.0001

Un improved drinking water source	1 [49]*	490, 543, 92, 492, 171, 198, 146, 95, 85, 358, 164, 138, 497, 521, 588, 458, 553, 278, 77, 629, 214, 318, 251, 573, 187, 239, 116, 22, 33, 568, 277, 527, 269, 556, 378, 630, 64, 439, 57, 480, 8, 210, 186, 454, 436, 566, 212, 501, 513	6.745502 N, 44.259011 E / 363.09 km	1140	743	2.03	240	<0.0001
Un improved toilet facilities	1 [238]*	253, 504, 612, 296, 258, 583, 268, 78, 98, 255, 528, 340, 188, 181, 638, 322, 584, 80, 425, 279, 312, 551, 640, 597, 400, 152, 156, 590, 636, 81, 327, 628, 84, 579, 52, 292, 479, 575, 45, 481, 355, 89, 461, 163, 604, 199, 538, 430, 542, 424, 598, 226, 404, 129, 158, 512, 169, 237, 94, 341, 550, 413, 132, 220, 605, 160, 392, 73, 623, 66, 384, 431, 99, 259, 298, 196, 117, 192, 136, 103, 143, 300, 516, 421, 602, 415, 449, 127, 263, 456, 79, 382, 362, 361, 167, 134, 627, 235, 128, 541, 511, 386, 442, 97, 351, 401, 585, 548, 403, 130, 172, 429, 38, 515, 24, 591, 478, 200, 545, 455, 488, 615, 109, 498, 249, 120, 3, 256, 533, 344, 332, 246, 559, 496, 176, 375, 544, 241, 206, 599, 410, 36, 354, 189, 474, 616, 389, 150, 137, 35, 183, 364, 571, 460, 482, 191, 611, 617, 348, 244, 494, 10, 18, 345, 531, 229, 184, 254, 267, 350, 218, 368, 457, 510, 427, 55, 320, 569, 324, 65, 88, 285, 570, 547, 209, 205, 409, 310, 572, 407, 124, 499, 178, 563, 335, 334, 621, 276, 595, 423, 620, 637, 581,	14.033877 N, 37.105922 E / 578.81 km	6150	5922	1.11	231	<0.0001

		433, 70, 508, 203, 161, 317, 349, 6, 294, 283, 517, 165, 399, 416, 17, 280, 440, 632, 295, 102, 304, 596, 234, 374, 201, 37, 485, 135, 624, 462, 4, 484, 395, 366, 75						

*Primary Clusters.
